# The complete chloroplast genome and phylogenetic analysis of *Primula stenocalyx* Maxim.

**DOI:** 10.1080/23802359.2021.1991245

**Published:** 2021-10-15

**Authors:** Yupeng Guo, Li Ma, Junqiao Li

**Affiliations:** Qinghai Provincial Key Laboratory of High Value Utilization of Characteristic Economic Plants, The College of Ecological Environment and Resources, Qinghai Minzu University, Xining, Qinghai, P. R. China

**Keywords:** *Primula stenocalyx* Maxim., chloroplast genome, phylogenetic analysis

## Abstract

*Primula stenocalyx* Maxim. is a perennial herb with purple umbel flowers. This alpine plant can survive at altitudes of 2700–4300 m. To explore the chloroplast genome, total DNA was extracted from a sample and sequenced, the reads of the chloroplast genome were assembled and annotated. The chloroplast genome of this plant has a circular form with a length of 153,678 bp comprising a large single-copy region (LSC, 84,236 bp), a small single-copy region (SSC, 17,746 bp), and two inverted repeats (IRs, 25,848 bp). The genome has a GC content of 37%. A total of 133 genes were predicted to contain 88 encoded proteins, 8 rRNAs and 37 tRNAs. The evolutionary history indicates that *P. stenocalyx* was grouped within *Primula* and formed a clade with *Primula knuthiana* with a 100% bootstrap support value. The complete cp genome of *P. stenocalyx* can serve as a reference for future studies on molecular biology, evolution, population genetics, taxonomy and resource protection.

*Primula stenocalyx* Maxim., belonging to Primulaceae, is a perennial herb with rosette leaves, typically indistinct petioles, and little purple umbel flowers (Hu and Kelso 1996). This plant is always found on south-facing grassy slopes, ditches, and peat and is widely distributed in Gansu, Sichuan Province and the Qinghai-Tibetan Plateau in China. The plant can survive at altitudes of 2700–4300 m (Chen and Hu 1990). Elumeeva has reported the leaf functional traits of this plant (Elumeeva et al. 2015). Here, we will report the complete chloroplast (cp) genome and analyze its phylogenetic relationship with other related species.

Several samples were collected from the Qilian Mountains (36°52’46"N, 102°17’15"E, Huzhu County) in Qinghai Province, and a specimen was deposited at the College of Ecological Environment and Resources, Qinghai Nationalities University (https://shxy.qhmu.edu.cn/, Junqiao Li, email: ljqlily2002@126.com) under voucher number HCEERQNU-20200516001. Total DNA was extracted from the fresh leaves of a sample with a Rapid Plant Genomic DNA Isolation Kit (Sangon Biotech (Shanghai) Co., Ltd.). Paired-end libraries with an average length of 500 bp were constructed and sequenced on the Illumina HiSeq4000 platform (Sangon Biotech (Shanghai) Co., Ltd.). The complete cp genome was assembled via NOVOPlasty 3.7.2 (Dierckxsens et al. 2017) with *Primula knuthiana* (GenBank accession no. NC_039350.1) as the reference genome and annotated via PGA (Qu et al. 2019).

The complete cp genome of *P. stenocalyx* (GenBank accession no. MW678839.1) has a typical quadripartite form with a length of 153,678 bp and is composed of a large single-copy region (LSC, 84,236 bp), a small single-copy region (SSC, 17,746 bp), and two inverted repeats (IR, 25,848 bp). The genome has a GC content of 37%. A total of 133 genes were predicted for this cp genome, comprising 88 encoded proteins, 8 rRNAs and 37 tRNAs.

Phylogenetic analysis was performed on the complete cp genomes of *P. stenocalyx* and 28 other related species in *Primula*, with three species in *Androsace* as the outgroup. The genome-wide alignment was constructed by HomBlocks (Bi et al. 2018), the evolutionary history was inferred using the maximum likelihood (ML) method by IQ-TREE 1.6.12 with the TVM + F+R3 model (Nguyen et al. 2015; Kalyaanamoorthy et al. 2017), bootstrap (BS) values were calculated by UFBoot2 from 1000 replicates (Hoang et al. 2018), and the final output file was edited in MEGA X (Kumar et al. 2018). As expected, *P. stenocalyx* was grouped within *Primula* and formed a clade with *Primula knuthiana* with a 100% BS support value (Figure 1). The complete cp genome of *P. stenocalyx* can serve as a reference for future studies on molecular biology, evolution, population genetics, taxonomy and resource protection.

**Figure 1.**
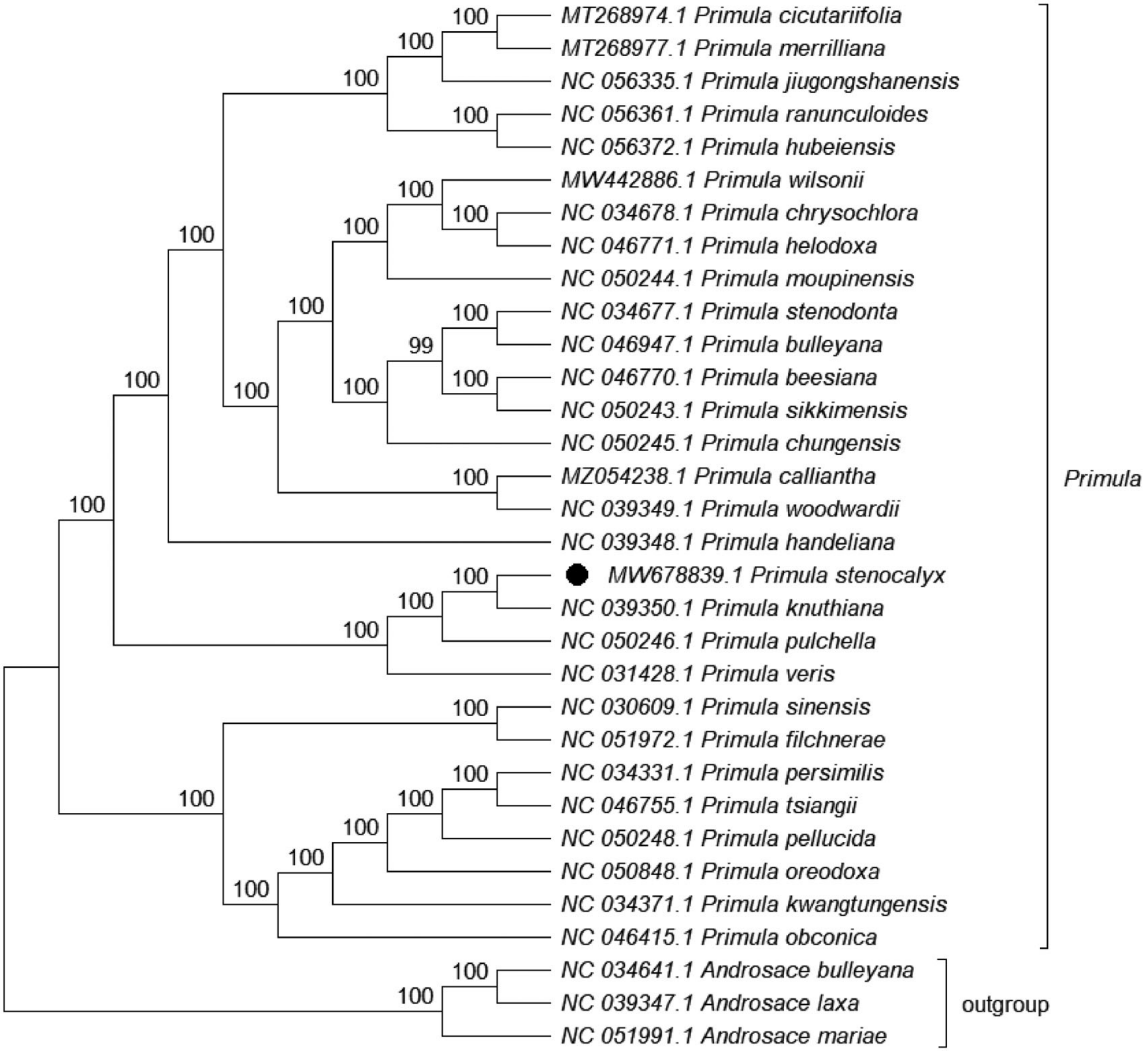
ML phylogenetic tree based on 32 species chloroplast genomes was constructed using IQ-TREE 1.6.12. Numbers on each node are bootstrap support values from 1000 replicates.

## Data Availability

The genome sequence data that support the findings of this study are openly available in GenBank of NCBI at (https://www.ncbi.nlm.nih.gov/nuccore/MW678839) under the accession no. MW678839. The associated BioProject, SRA, and Bio-Sample numbers are PRJNA 725315, SRR 14361579, and SAMN 18875961, respectively.
